# (*N*-Benzoyl-*N*′-phenyl­thio­urea-κ*S*)chlorido(η^4^-1,5-cyclo­octa­diene)rhodium(I)

**DOI:** 10.1107/S1600536810029740

**Published:** 2010-07-31

**Authors:** P. D. Riekert Kotze, Andreas Roodt, Johan A. Venter, Stefanus Otto

**Affiliations:** a Department of Chemistry, University of Free State, Bloemfontein 9300, South Africa

## Abstract

The title compound, [RhCl(C_8_H_12_)(C_14_H_12_N_2_OS)], is a rhodium(I) derivative with a functionalized thio­urea ligand. Despite the presence of several heteroatoms, the thio­urea ligand coordinates only in a monodentate fashion *via* the S atom. The geometry of the coordination sphere is approximately square planar about the Rh^I^ atom, with two bonds to the π-electrons of the 1,5-cyclo­octa­diene ligand, one bond to the Cl^−^ ligand and one bond to the S atom of the thio­urea ligand. The mol­ecular structure is stabilized by intra­molecular N—H⋯O and N—H⋯Cl hydrogen bonding. Inter­molecular N—H⋯O hydrogen-bonding inter­actions lead to the formation of layers extending parallel to (011).

## Related literature

For related Rh(I) complexes containing thio­urea ligands, see: Cauzzi *et al.* (1995 ▶); Kemp *et al.* (1996 ▶, 1997 ▶); Roodt *et al.* (1994 ▶). For related Rh(I) complexes containing other or similar β-diketones and π-bonding ligands, see: Bahl *et al.* (2000 ▶); Brink *et al.* (2007**a* ▶,b* ▶); Leipoldt *et al.* (1977 ▶, 1980 ▶); Roodt *et al.* (2003 ▶); Steyl *et al.* (2004 ▶). For structural data for the thio­urea ligand *N*-phenyl-*N*′-benzoyl­thio­urea, see: Yamin & Yusof (2003 ▶).
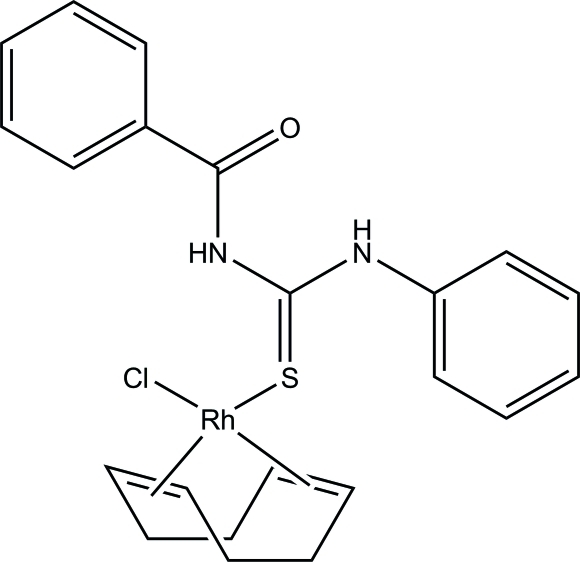


## Experimental

### 

#### Crystal data

[RhCl(C_8_H_12_)(C_14_H_12_N_2_OS)]
*M* *_r_* = 502.85
Triclinic, 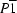

*a* = 6.6703 (2) Å
*b* = 10.1665 (4) Å
*c* = 14.9616 (5) Å
α = 96.891 (2)°
β = 91.588 (2)°
γ = 90.616 (2)°
*V* = 1006.78 (6) Å^3^
*Z* = 2
Mo *K*α radiation
μ = 1.10 mm^−1^
*T* = 100 K
0.18 × 0.17 × 0.08 mm

#### Data collection

Bruker APEXII CCD area-detector diffractometer
Absorption correction: multi-scan (*SADABS*; Bruker, 2001 ▶) *T*_min_ = 0.827, *T*_max_ = 0.917
18721 measured reflections
4980 independent reflections
4476 reflections with *I* > 2σ(*I*)
*R*_int_ = 0.032

#### Refinement

*R*[*F* ^2^ > 2σ(*F* ^2^)] = 0.029
*wR*(*F* ^2^) = 0.103
*S* = 1.23
4980 reflections
253 parameters
H-atom parameters constrained
Δρ_max_ = 0.77 e Å^−3^
Δρ_min_ = −0.66 e Å^−3^

### 

Data collection: *APEX2* (Bruker, 2005 ▶); cell refinement: *SAINT-Plus* (Bruker, 2004 ▶); data reduction: *SAINT-Plus* and *XPREP* (Bruker, 2004 ▶); program(s) used to solve structure: *SHELXS97* (Sheldrick, 2008 ▶); program(s) used to refine structure: *SHELXL97* (Sheldrick, 2008 ▶); molecular graphics: *Mercury* (Macrae *et al.*, 2006 ▶); software used to prepare material for publication: *WinGX* (Farrugia, 1999 ▶) and *publCIF* (Westrip, 2010 ▶).

## Supplementary Material

Crystal structure: contains datablocks I, global. DOI: 10.1107/S1600536810029740/wm2369sup1.cif

Structure factors: contains datablocks I. DOI: 10.1107/S1600536810029740/wm2369Isup2.hkl

Additional supplementary materials:  crystallographic information; 3D view; checkCIF report

## Figures and Tables

**Table 1:** Selected bond lengths (Å)

Rh1—C15	2.105 (3)
Rh1—C22	2.121 (3)
Rh1—C19	2.144 (3)
Rh1—C18	2.170 (3)
Rh1—S1	2.3803 (7)
Rh1—Cl1	2.3850 (7)

**Table 2:** Hydrogen-bond geometry (Å, °)

*D*—H⋯*A*	*D*—H	H⋯*A*	*D*⋯*A*	*D*—H⋯*A*
N2—H2⋯O1	0.88	1.99	2.657 (3)	132
N2—H2⋯O1^i^	0.88	2.32	3.053 (3)	141
N1—H1⋯Cl1	0.88	2.47	3.253 (3)	148

Symmetry code: (i) 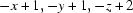.

**Fig. 1.**
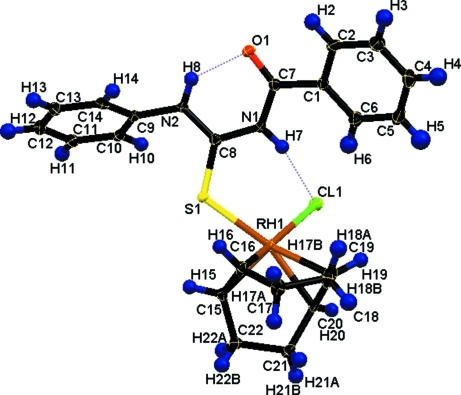
: A representation of the title compound, displaying the numbering scheme and displacement ellipsoids at the 50% probability level.

## supplementary crystallographic information

### Comment

Rhodium complexes containing σ-bonding bidentate ligands such as β-diketones and π-bonding ligands such as arenes, carbonyls *etc*. simultaneously, are a well known compounds. For a few examples, see:  Bahl *et al.* (2000), Brink *et al.* (2007*a*,*b*), Cauzzi *et al.* (1995), Leipoldt *et al.* (1977, 1980), Roodt *et al.* (2003), Steyl *et al.* (2004).

The title compound, [Rh(C_8_H_12_)(C_14_H_12_N_2_OS)Cl], features a functionalized thiourea ligand, which has been shown by previous authors (Kemp *et al.*, 1996, 1997; Roodt *et al.*, 1994)) to have the ability to co-ordinate in a bidentate fashion as many other hetero-atom bidentate ligands do, including β-diketones and derivatives. However, in the title compound this ligand only co-ordinates in a monodentate fashion *via* the sulfur atom to the rhodium atom.

The rhodium(I) complex is found to have a slightly distorted square-planar coordination about the rhodium centre with a chlorine atom *cis* to the sulfur atom (Fig. 1). The packing of the complex is well established by the presence of intra- and intermolecular hydrogen bonding. Intramolecular hydrogen bonding occurs between O1 and N2 with a distance of 1.99 Å. The same observation was made with the free ligand (Yamin & Yusof, 2003). This interaction suggests the prefered orientation of the free ligand to have its oxygen *trans* to the sulfur atom and it clearly translates to the orientation found in the title compound. Hydrogen bonding was also observed between the nitrogen atom N1 and the chlorine atom Cl1, with a distance of 2.47 Å, which added onto the effect of stabilizing the orientation found in the title compound. Since two molecules are orientated about an inversion centre, the oxygen atom O1 as well as the nitrogen atom N2 were found in close approximation to the oxygen atom in the next molecule. As a result, intermolecular hydrogen bonding between the two oxygen atoms as well as between N2 and O1 were established with distances of 2.980 Å and 3.053 Å, respectively. The intermolecular hydrogen bonding leads to a layered assembly of the molecules, extending approximately parallel to (011).

In addition, a vast variety of short contacts *via* van der Waals interactions are found to be present amongst various atoms. These short contacts are suspected to be the cause of the distortion found in the *cyclo*-octadiene ring as six of its atoms are pulled in various directions.

### Experimental

Dichloridodicyclo-octadienedirhodium(I) (20.0 mg, 0.0406 mmol) was allowed to react with *N*-phenyl-*N*'-benzoylthiourea (20.8 mg, 0.0406 mmol) in acetone (2 cm^3^). Upon evaporation yellow crystals were obtained.

### Refinement

The aliphatic as well as aromatic H atoms were placed in geometrically idealized positions and constrained to ride on their parent atoms with *U*_iso_(H) = 1.2Ueq(C). The highest residual electron density was located 0.79 Å from C15 and the deepest hole was 0.88 Å from Rh1.

### Crystal data

**Table:**

[RhCl(C_8_H_12_)(C_14_H_12_N_2_OS)]	*Z* = 2
*M**_r_* = 502.85	*F*(000) = 512
Triclinic, *P*1	*D*_x_ = 1.659 Mg m^−^^3^
Hall symbol: -P 1	Mo *K*α radiation, λ = 0.71073 Å
*a* = 6.6703 (2) Å	Cell parameters from 8458 reflections
*b* = 10.1665 (4) Å	θ = 3.1–28.3°
*c* = 14.9616 (5) Å	µ = 1.10 mm^−^^1^
α = 96.891 (2)°	*T* = 100 K
β = 91.588 (2)°	Platelet, yellow
γ = 90.616 (2)°	0.18 × 0.17 × 0.08 mm
*V* = 1006.78 (6) Å^3^	

### Data collection

**Table:**

Bruker APEXII CCD area-detector diffractometer	4980 independent reflections
Radiation source: fine-focus sealed tube	4476 reflections with *I* > 2σ(*I*)
graphite	*R*_int_ = 0.032
φ– and ω–scans	θ_max_ = 28.3°, θ_min_ = 1.4°
Absorption correction: multi-scan (*SADABS*; Bruker, 2001)	*h* = −8→8
*T*_min_ = 0.827, *T*_max_ = 0.917	*k* = −13→13
18721 measured reflections	*l* = −19→19

### Refinement

**Table:**

Refinement on *F*^2^	Primary atom site location: structure-invariant direct methods
Least-squares matrix: full	Secondary atom site location: difference Fourier map
*R*[*F*^2^ > 2σ(*F*^2^)] = 0.029	Hydrogen site location: inferred from neighbouring sites
*wR*(*F*^2^) = 0.103	H-atom parameters constrained
*S* = 1.23	*w* = 1/[σ^2^(*F*_o_^2^) + (0.0511*P*)^2^ + 0.7134*P*] where *P* = (*F*_o_^2^ + 2*F*_c_^2^)/3
4980 reflections	(Δ/σ)_max_ = 0.001
253 parameters	Δρ_max_ = 0.77 e Å^−^^3^
0 restraints	Δρ_min_ = −0.66 e Å^−^^3^

### Special details

**Table:**

Geometry. All e.s.d.'s (except the e.s.d. in the dihedral angle between two l.s. planes) are estimated using the full covariance matrix. The cell e.s.d.'s are taken into account individually in the estimation of e.s.d.'s in distances, angles and torsion angles; correlations between e.s.d.'s in cell parameters are only used when they are defined by crystal symmetry. An approximate (isotropic) treatment of cell e.s.d.'s is used for estimating e.s.d.'s involving l.s. planes.
Refinement. Refinement of *F*^2^ against ALL reflections. The weighted *R*-factor *wR* and goodness of fit *S* are based on *F*^2^, conventional *R*-factors *R* are based on *F*, with *F* set to zero for negative *F*^2^. The threshold expression of *F*^2^ > σ(*F*^2^) is used only for calculating *R*-factors(gt) *etc*. and is not relevant to the choice of reflections for refinement. *R*-factors based on *F*^2^ are statistically about twice as large as those based on *F*, and *R*- factors based on ALL data will be even larger.

### Fractional atomic coordinates and isotropic or equivalent isotropic displacement parameters (Å^2^)

**Table:**

	*x*	*y*	*z*	*U*_iso_*/*U*_eq_	
Rh1	0.63173 (3)	0.78913 (2)	0.637367 (14)	0.01158 (9)	
Cl1	0.56345 (11)	0.95960 (7)	0.75606 (5)	0.01837 (16)	
S1	0.42173 (10)	0.62944 (7)	0.69410 (5)	0.01483 (15)	
O1	0.6682 (3)	0.5834 (2)	0.97351 (14)	0.0187 (4)	
N2	0.3860 (3)	0.5223 (2)	0.84601 (16)	0.0155 (5)	
H2	0.4237	0.5172	0.9023	0.019*	
N1	0.6287 (3)	0.6887 (2)	0.84822 (16)	0.0147 (5)	
H1	0.6658	0.7598	0.8243	0.018*	
C9	0.2314 (4)	0.4322 (3)	0.80693 (18)	0.0135 (5)	
C8	0.4760 (4)	0.6125 (3)	0.80349 (19)	0.0134 (5)	
C10	0.0498 (4)	0.4796 (3)	0.77768 (19)	0.0148 (5)	
H10	0.0289	0.5722	0.7802	0.018*	
C6	1.0460 (4)	0.6965 (3)	1.0142 (2)	0.0201 (6)	
H6	1.0027	0.6260	1.0457	0.024*	
C12	−0.0712 (4)	0.2545 (3)	0.7407 (2)	0.0195 (6)	
H12	−0.1754	0.1938	0.7186	0.023*	
C1	0.9224 (4)	0.7406 (3)	0.94780 (19)	0.0142 (5)	
C7	0.7298 (4)	0.6651 (3)	0.92667 (19)	0.0139 (5)	
C3	1.1740 (5)	0.9028 (3)	0.9235 (2)	0.0290 (8)	
H3	1.2182	0.9737	0.8926	0.035*	
C11	−0.1017 (4)	0.3899 (3)	0.7445 (2)	0.0173 (6)	
H11	−0.2265	0.4217	0.7244	0.021*	
C2	0.9856 (5)	0.8446 (3)	0.9022 (2)	0.0235 (7)	
H2A	0.9014	0.8756	0.8572	0.028*	
C14	0.2642 (4)	0.2972 (3)	0.80220 (19)	0.0171 (6)	
H14	0.3900	0.2657	0.8214	0.021*	
C13	0.1126 (4)	0.2078 (3)	0.7693 (2)	0.0183 (6)	
H13	0.1343	0.1152	0.7664	0.022*	
C19	0.8922 (4)	0.8992 (3)	0.6073 (2)	0.0167 (6)	
H19	0.9299	0.9740	0.6547	0.020*	
C16	0.5920 (5)	0.7470 (3)	0.4355 (2)	0.0183 (6)	
H16A	0.4513	0.7729	0.4255	0.022*	
H16B	0.6341	0.6913	0.3806	0.022*	
C5	1.2327 (5)	0.7560 (3)	1.0342 (2)	0.0233 (7)	
H5	1.3172	0.7259	1.0795	0.028*	
C4	1.2967 (5)	0.8581 (3)	0.9890 (2)	0.0251 (7)	
H4	1.4251	0.8978	1.0029	0.030*	
C20	1.0617 (4)	0.8056 (3)	0.5829 (2)	0.0225 (7)	
H20A	1.1359	0.7893	0.6386	0.027*	
H20B	1.1559	0.8481	0.5448	0.027*	
C21	0.9898 (4)	0.6720 (3)	0.5324 (2)	0.0196 (6)	
H21A	0.9898	0.6774	0.4668	0.023*	
H21B	1.0847	0.6022	0.5456	0.023*	
C22	0.7794 (4)	0.6336 (3)	0.5590 (2)	0.0153 (6)	
H22	0.7719	0.5465	0.5834	0.018*	
C18	0.7382 (4)	0.9284 (3)	0.54959 (19)	0.0147 (5)	
H18	0.6844	1.0199	0.5628	0.018*	
C15	0.6023 (4)	0.6668 (3)	0.51368 (19)	0.0150 (6)	
H15	0.4931	0.5983	0.5121	0.018*	
C17	0.7254 (4)	0.8724 (3)	0.4506 (2)	0.0180 (6)	
H17A	0.8617	0.8509	0.4294	0.022*	
H17B	0.6698	0.9402	0.4149	0.022*	

### Atomic displacement parameters (Å^2^)

**Table:**

	*U*^11^	*U*^22^	*U*^33^	*U*^12^	*U*^13^	*U*^23^
Rh1	0.01211 (12)	0.01013 (13)	0.01270 (13)	−0.00102 (8)	0.00040 (8)	0.00221 (9)
Cl1	0.0245 (3)	0.0138 (3)	0.0166 (3)	0.0010 (3)	0.0037 (3)	0.0002 (3)
S1	0.0163 (3)	0.0168 (3)	0.0120 (3)	−0.0051 (3)	−0.0012 (2)	0.0050 (3)
O1	0.0183 (10)	0.0233 (11)	0.0155 (10)	−0.0071 (8)	−0.0018 (8)	0.0075 (9)
N2	0.0171 (11)	0.0185 (12)	0.0114 (11)	−0.0064 (9)	−0.0025 (9)	0.0045 (10)
N1	0.0167 (11)	0.0141 (12)	0.0136 (12)	−0.0049 (9)	−0.0015 (9)	0.0044 (9)
C9	0.0151 (12)	0.0139 (13)	0.0113 (13)	−0.0044 (10)	0.0008 (10)	0.0015 (10)
C8	0.0132 (12)	0.0139 (13)	0.0127 (13)	0.0002 (10)	0.0008 (10)	0.0005 (10)
C10	0.0177 (13)	0.0135 (13)	0.0139 (14)	−0.0007 (10)	0.0045 (10)	0.0031 (11)
C6	0.0179 (13)	0.0270 (16)	0.0168 (15)	−0.0051 (12)	0.0011 (11)	0.0086 (12)
C12	0.0219 (14)	0.0180 (15)	0.0181 (15)	−0.0077 (11)	−0.0011 (11)	0.0013 (12)
C1	0.0131 (12)	0.0149 (14)	0.0141 (13)	−0.0023 (10)	0.0012 (10)	0.0001 (11)
C7	0.0132 (12)	0.0152 (14)	0.0130 (13)	−0.0011 (10)	0.0006 (10)	0.0009 (11)
C3	0.0285 (17)	0.0246 (17)	0.035 (2)	−0.0126 (14)	−0.0060 (14)	0.0125 (15)
C11	0.0144 (12)	0.0212 (15)	0.0167 (14)	−0.0019 (11)	−0.0028 (11)	0.0041 (12)
C2	0.0230 (15)	0.0233 (16)	0.0249 (17)	−0.0072 (12)	−0.0068 (12)	0.0085 (13)
C14	0.0191 (13)	0.0188 (15)	0.0135 (14)	−0.0010 (11)	−0.0029 (11)	0.0034 (11)
C13	0.0236 (14)	0.0141 (14)	0.0172 (14)	−0.0024 (11)	−0.0044 (11)	0.0036 (11)
C19	0.0140 (12)	0.0126 (13)	0.0238 (15)	−0.0043 (10)	0.0036 (11)	0.0023 (11)
C16	0.0250 (14)	0.0167 (14)	0.0132 (14)	−0.0025 (11)	0.0014 (11)	0.0024 (11)
C5	0.0186 (14)	0.0278 (17)	0.0243 (16)	−0.0010 (12)	−0.0049 (12)	0.0080 (13)
C4	0.0171 (14)	0.0284 (18)	0.0291 (18)	−0.0070 (12)	−0.0017 (12)	0.0023 (14)
C20	0.0120 (13)	0.0177 (15)	0.0373 (19)	−0.0008 (11)	0.0024 (12)	0.0008 (13)
C21	0.0142 (13)	0.0163 (15)	0.0281 (17)	0.0030 (11)	0.0040 (11)	0.0014 (12)
C22	0.0178 (13)	0.0090 (13)	0.0186 (14)	−0.0003 (10)	0.0037 (11)	−0.0005 (11)
C18	0.0178 (12)	0.0088 (12)	0.0180 (14)	−0.0039 (10)	0.0025 (11)	0.0041 (11)
C15	0.0185 (13)	0.0116 (13)	0.0144 (14)	−0.0039 (10)	0.0010 (10)	−0.0006 (11)
C17	0.0212 (14)	0.0145 (14)	0.0196 (15)	−0.0020 (11)	0.0041 (11)	0.0061 (12)

### Geometric parameters (Å, °)

**Table:**

Rh1—C15	2.105 (3)	C11—H11	0.9500
Rh1—C22	2.121 (3)	C2—H2A	0.9500
Rh1—C19	2.144 (3)	C14—C13	1.391 (4)
Rh1—C18	2.170 (3)	C14—H14	0.9500
Rh1—S1	2.3803 (7)	C13—H13	0.9500
Rh1—Cl1	2.3850 (7)	C19—C18	1.382 (4)
S1—C8	1.696 (3)	C19—C20	1.510 (4)
O1—C7	1.224 (3)	C19—H19	1.0000
N2—C8	1.325 (4)	C16—C15	1.504 (4)
N2—C9	1.436 (3)	C16—C17	1.538 (4)
N2—H2	0.8800	C16—H16A	0.9900
N1—C8	1.381 (3)	C16—H16B	0.9900
N1—C7	1.385 (4)	C5—C4	1.375 (5)
N1—H1	0.8800	C5—H5	0.9500
C9—C14	1.386 (4)	C4—H4	0.9500
C9—C10	1.388 (4)	C20—C21	1.537 (4)
C10—C11	1.393 (4)	C20—H20A	0.9900
C10—H10	0.9500	C20—H20B	0.9900
C6—C5	1.388 (4)	C21—C22	1.528 (4)
C6—C1	1.392 (4)	C21—H21A	0.9900
C6—H6	0.9500	C21—H21B	0.9900
C12—C11	1.388 (4)	C22—C15	1.410 (4)
C12—C13	1.394 (4)	C22—H22	1.0000
C12—H12	0.9500	C18—C17	1.521 (4)
C1—C2	1.393 (4)	C18—H18	1.0000
C1—C7	1.496 (4)	C15—H15	1.0000
C3—C4	1.382 (5)	C17—H17A	0.9900
C3—C2	1.395 (4)	C17—H17B	0.9900
C3—H3	0.9500		
			
C15—Rh1—C22	38.97 (11)	C14—C13—H13	120.1
C15—Rh1—C19	97.74 (11)	C12—C13—H13	120.1
C22—Rh1—C19	82.06 (11)	C18—C19—C20	125.5 (3)
C15—Rh1—C18	81.32 (11)	C18—C19—Rh1	72.36 (16)
C22—Rh1—C18	89.91 (11)	C20—C19—Rh1	109.79 (19)
C19—Rh1—C18	37.35 (11)	C18—C19—H19	113.8
C15—Rh1—S1	85.42 (8)	C20—C19—H19	113.8
C22—Rh1—S1	89.46 (8)	Rh1—C19—H19	113.8
C19—Rh1—S1	161.71 (9)	C15—C16—C17	112.8 (2)
C18—Rh1—S1	159.60 (8)	C15—C16—H16A	109.0
C15—Rh1—Cl1	160.03 (8)	C17—C16—H16A	109.0
C22—Rh1—Cl1	160.91 (8)	C15—C16—H16B	109.0
C19—Rh1—Cl1	89.02 (8)	C17—C16—H16B	109.0
C18—Rh1—Cl1	93.19 (8)	H16A—C16—H16B	107.8
S1—Rh1—Cl1	94.05 (2)	C4—C5—C6	120.6 (3)
C8—S1—Rh1	112.67 (10)	C4—C5—H5	119.7
C8—N2—C9	124.8 (2)	C6—C5—H5	119.7
C8—N2—H2	117.6	C5—C4—C3	119.9 (3)
C9—N2—H2	117.6	C5—C4—H4	120.0
C8—N1—C7	126.9 (2)	C3—C4—H4	120.0
C8—N1—H1	116.5	C19—C20—C21	113.1 (2)
C7—N1—H1	116.5	C19—C20—H20A	109.0
C14—C9—C10	120.7 (3)	C21—C20—H20A	109.0
C14—C9—N2	118.8 (3)	C19—C20—H20B	109.0
C10—C9—N2	120.5 (3)	C21—C20—H20B	109.0
N2—C8—N1	118.5 (3)	H20A—C20—H20B	107.8
N2—C8—S1	122.2 (2)	C22—C21—C20	112.1 (2)
N1—C8—S1	119.1 (2)	C22—C21—H21A	109.2
C9—C10—C11	119.3 (3)	C20—C21—H21A	109.2
C9—C10—H10	120.3	C22—C21—H21B	109.2
C11—C10—H10	120.3	C20—C21—H21B	109.2
C5—C6—C1	119.6 (3)	H21A—C21—H21B	107.9
C5—C6—H6	120.2	C15—C22—C21	123.7 (3)
C1—C6—H6	120.2	C15—C22—Rh1	69.93 (16)
C11—C12—C13	119.9 (3)	C21—C22—Rh1	113.40 (19)
C11—C12—H12	120.1	C15—C22—H22	114.0
C13—C12—H12	120.1	C21—C22—H22	114.0
C6—C1—C2	120.0 (3)	Rh1—C22—H22	114.0
C6—C1—C7	115.9 (3)	C19—C18—C17	122.9 (3)
C2—C1—C7	123.9 (3)	C19—C18—Rh1	70.29 (16)
O1—C7—N1	121.6 (2)	C17—C18—Rh1	112.65 (18)
O1—C7—C1	122.5 (3)	C19—C18—H18	114.4
N1—C7—C1	115.9 (2)	C17—C18—H18	114.4
C4—C3—C2	120.5 (3)	Rh1—C18—H18	114.4
C4—C3—H3	119.7	C22—C15—C16	125.6 (2)
C2—C3—H3	119.7	C22—C15—Rh1	71.11 (16)
C12—C11—C10	120.4 (3)	C16—C15—Rh1	111.48 (19)
C12—C11—H11	119.8	C22—C15—H15	113.7
C10—C11—H11	119.8	C16—C15—H15	113.7
C1—C2—C3	119.2 (3)	Rh1—C15—H15	113.7
C1—C2—H2A	120.4	C18—C17—C16	111.3 (2)
C3—C2—H2A	120.4	C18—C17—H17A	109.4
C9—C14—C13	119.8 (3)	C16—C17—H17A	109.4
C9—C14—H14	120.1	C18—C17—H17B	109.4
C13—C14—H14	120.1	C16—C17—H17B	109.4
C14—C13—C12	119.9 (3)	H17A—C17—H17B	108.0
			
C15—Rh1—S1—C8	147.13 (13)	C18—C19—C20—C21	−47.9 (4)
C22—Rh1—S1—C8	108.32 (13)	Rh1—C19—C20—C21	34.1 (3)
C19—Rh1—S1—C8	46.3 (3)	C19—C20—C21—C22	−29.6 (4)
C18—Rh1—S1—C8	−163.4 (2)	C20—C21—C22—C15	91.3 (4)
Cl1—Rh1—S1—C8	−52.90 (11)	C20—C21—C22—Rh1	10.6 (3)
C8—N2—C9—C14	120.1 (3)	C19—Rh1—C22—C15	−112.87 (18)
C8—N2—C9—C10	−62.3 (4)	C18—Rh1—C22—C15	−76.23 (17)
C9—N2—C8—N1	−177.1 (2)	S1—Rh1—C22—C15	83.38 (15)
C9—N2—C8—S1	−1.8 (4)	Cl1—Rh1—C22—C15	−175.75 (18)
C7—N1—C8—N2	14.4 (4)	C15—Rh1—C22—C21	119.1 (3)
C7—N1—C8—S1	−161.1 (2)	C19—Rh1—C22—C21	6.2 (2)
Rh1—S1—C8—N2	−175.2 (2)	C18—Rh1—C22—C21	42.8 (2)
Rh1—S1—C8—N1	0.1 (2)	S1—Rh1—C22—C21	−157.6 (2)
C14—C9—C10—C11	1.0 (4)	Cl1—Rh1—C22—C21	−56.7 (4)
N2—C9—C10—C11	−176.5 (3)	C20—C19—C18—C17	−2.7 (4)
C5—C6—C1—C2	−0.5 (5)	Rh1—C19—C18—C17	−104.8 (2)
C5—C6—C1—C7	176.2 (3)	C20—C19—C18—Rh1	102.1 (3)
C8—N1—C7—O1	−12.9 (4)	C15—Rh1—C18—C19	−115.12 (18)
C8—N1—C7—C1	164.3 (3)	C22—Rh1—C18—C19	−76.96 (18)
C6—C1—C7—O1	11.1 (4)	S1—Rh1—C18—C19	−165.15 (18)
C2—C1—C7—O1	−172.3 (3)	Cl1—Rh1—C18—C19	84.20 (16)
C6—C1—C7—N1	−166.1 (3)	C15—Rh1—C18—C17	3.3 (2)
C2—C1—C7—N1	10.5 (4)	C22—Rh1—C18—C17	41.4 (2)
C13—C12—C11—C10	−0.7 (4)	C19—Rh1—C18—C17	118.4 (3)
C9—C10—C11—C12	−0.1 (4)	S1—Rh1—C18—C17	−46.8 (3)
C6—C1—C2—C3	0.5 (5)	Cl1—Rh1—C18—C17	−157.41 (19)
C7—C1—C2—C3	−175.9 (3)	C21—C22—C15—C16	−2.0 (4)
C4—C3—C2—C1	−0.1 (5)	Rh1—C22—C15—C16	103.4 (3)
C10—C9—C14—C13	−1.1 (4)	C21—C22—C15—Rh1	−105.3 (3)
N2—C9—C14—C13	176.4 (3)	C17—C16—C15—C22	−46.0 (4)
C9—C14—C13—C12	0.4 (4)	C17—C16—C15—Rh1	35.6 (3)
C11—C12—C13—C14	0.5 (4)	C19—Rh1—C15—C22	67.06 (18)
C15—Rh1—C19—C18	64.59 (18)	C18—Rh1—C15—C22	100.73 (17)
C22—Rh1—C19—C18	100.38 (18)	S1—Rh1—C15—C22	−94.81 (15)
S1—Rh1—C19—C18	163.5 (2)	Cl1—Rh1—C15—C22	175.93 (17)
Cl1—Rh1—C19—C18	−96.55 (16)	C22—Rh1—C15—C16	−121.7 (3)
C15—Rh1—C19—C20	−57.6 (2)	C19—Rh1—C15—C16	−54.7 (2)
C22—Rh1—C19—C20	−21.8 (2)	C18—Rh1—C15—C16	−21.0 (2)
C18—Rh1—C19—C20	−122.2 (3)	S1—Rh1—C15—C16	143.47 (19)
S1—Rh1—C19—C20	41.2 (4)	Cl1—Rh1—C15—C16	54.2 (3)
Cl1—Rh1—C19—C20	141.2 (2)	C19—C18—C17—C16	95.1 (3)
C1—C6—C5—C4	0.0 (5)	Rh1—C18—C17—C16	14.6 (3)
C6—C5—C4—C3	0.4 (5)	C15—C16—C17—C18	−32.7 (3)
C2—C3—C4—C5	−0.4 (5)		

### Hydrogen-bond geometry (Å, °)

**Table:**

*D*—H···*A*	*D*—H	H···*A*	*D*···*A*	*D*—H···*A*
N2—H2···O1	0.88	1.99	2.657 (3)	132
N2—H2···O1^i^	0.88	2.32	3.053 (3)	141
N1—H1···Cl1	0.88	2.47	3.253 (3)	148

Symmetry codes: (i) −*x*+1, −*y*+1, −*z*+2.
